# The Complete Chloroplast Genome of *Capsicum annuum* var. *glabriusculum* Using Illumina Sequencing

**DOI:** 10.3390/molecules200713080

**Published:** 2015-07-20

**Authors:** Sebastin Raveendar, Young-Wang Na, Jung-Ro Lee, Donghwan Shim, Kyung-Ho Ma, Sok-Young Lee, Jong-Wook Chung

**Affiliations:** 1 National Agrobiodiversity Center, National Academy of Agricultural Science, Rural Development Administration, Jeonju 560-500, Korea; E-Mails: raveendars@gmail.com (S.R.); ywna@korea.kr (Y.-W.N.); jrmail@korea.kr (J.-R.L.); khma@korea.kr (K.-H.M.); lsy007@korea.kr (S.-Y.L.); 2 Department of Forest Genetic Resources, Korea Forest Research Institute, Suwon 441-350, Korea; E-Mail: shim104@korea.kr

**Keywords:** *Capsicum*, bird pepper, chloroplast, DNA sequencing

## Abstract

Chloroplast (cp) genome sequences provide a valuable source for DNA barcoding. Molecular phylogenetic studies have concentrated on DNA sequencing of conserved gene loci. However, this approach is time consuming and more difficult to implement when gene organization differs among species. Here we report the complete re-sequencing of the cp genome of *Capsicum* pepper (*Capsicum annuum* var. *glabriusculum*) using the Illumina platform. The total length of the cp genome is 156,817 bp with a 37.7% overall GC content. A pair of inverted repeats (IRs) of 50,284 bp were separated by a small single copy (SSC; 18,948 bp) and a large single copy (LSC; 87,446 bp). The number of cp genes in *C. annuum* var. *glabriusculum* is the same as that in other *Capsicum* species. Variations in the lengths of LSC; SSC and IR regions were the main contributors to the size variation in the cp genome of this species. A total of 125 simple sequence repeat (SSR) and 48 insertions or deletions variants were found by sequence alignment of *Capsicum* cp genome. These findings provide a foundation for further investigation of cp genome evolution in *Capsicum* and other higher plants.

## 1. Introduction

Chloroplasts (cp) are membrane-bound organelles, mainly involved in the photosynthetic conversion of atmospheric CO_2_ into carbohydrates in which light energy is stored as chemical energy. Cp possess their own genome that encodes a range of genes, involved mainly in photosynthesis and some essential metabolic pathways [1,2]. The first reports on complete cp genome sequences from tobacco and liverwort were reported in 1986 [3,4]. Since then, with emerging rapid and cost-effective NGS sequencing approaches, 342 cp genome sequences from different lineages have been reported [5]. Analyses of the cp genome among land plants show that their genome structure and organization are highly conserved with a quadripartite structure [6,7,8].

*Capsicum* L. (pepper) is a genus of the highly diverse Solanaceae family and comprises approximately 32 recognized species [9]. *Capsicum* originated in the New World and is cultivated in temperate and tropical regions [10,11], but knowledge of its domestication is incomplete. *Capsicum annuum* var. *glabriusculum*, is a unique *Capsicum* species commonly known as the American bird pepper well-known for its rich variation in flavor and aroma. Peppers play important roles in various aspects of the economy, food and pharmaceutics [12]. Therefore, knowledge regarding the genetic diversity among the germplasm is vital for strategic germplasm collection, maintenance, conservation and utilization.

DNA barcoding is a taxonomic method that aims to provide rapid and accurate species identification using a standard DNA region. The highly conserved structure of cp genome organization is a potential source of information for phylogenetic reconstruction of species relationships among plants. The cp genome has a simple and stable genetic structure, and universal primers can be used to amplify target sequences. In land plants, the highly variable cp gene sequences, such as *matK*, *rbcL* and *psbA-trnH*, are considered efficient DNA barcodes [13,14,15]. The advent of DNA barcoding to identify plant species appears to be promising, but most of the individual plastid candidate barcodes lack species level resolution [16,17].

For finding multi-locus DNA barcodes of high resolution at species level, it is essential to determine the distribution and location of highly arranged sequences information present in the cp genome. Until now, only three complete cp genome sequences from *Capsicum* species, American bird pepper (*Capsicum annuum* var. *glabriusculum*) [18], Korean landrace “subicho” pepper (*Capsicum annuum* var. *annuum*) [19] and a cultivated pepper (*Capsicum annuum* L.) [20], have been reported. The complete cp genome sequence of *Capsicum* pepper, *C. annuum* var. *glabriusculum*, reported here augments the genetic information for *Capsicum* species which will facilitate multi-locus choice for plant barcoding, population, phylogenetic and cp genetic engineering studies of this species.

## 2. Results and Discussion

### 2.1. Chloroplast Genome Assembly

**Figure 1:**
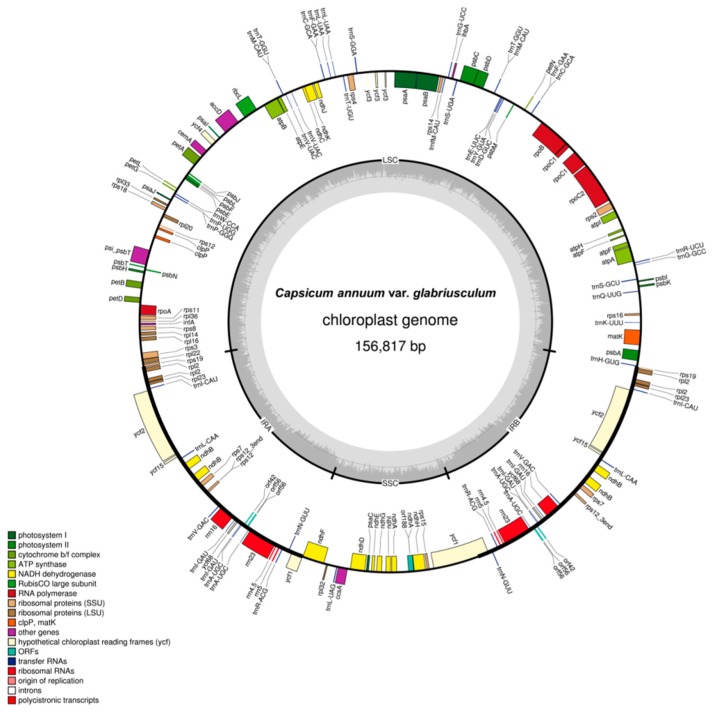
Complete chloroplast genome map of *C. annuum* var. *glabriusculum*. Genes drawn inside the circle are transcribed clockwise, and those outside are transcribed counterclockwise and marked by two arrows. Differential functional gene groups are color-coded. The GC content variation is shown in the middle circle.

We sequenced the cp genome of *C. annuum* var. *glabriusculum* using the Illumina genome analyzer platform. Illumina paired-end (2 × 300 bp) sequencing produced a total of 7,716,442 paired-end reads, with an average fragment length of 277 bp, which were then analyzed to generate 1,964,163,823 bp of sequence. Low quality reads (Q20) were filtered out, and the remaining high quality reads were mapped to the reference cp genome of *Capsicum*, which contains 29,609,440 mapped nucleotides with an average coverage of 188× on the cp genome. The cp reads extracted from the Illumina dataset were assembled into a total of four contigs. Contig alignment and scaffolding based on paired-end data resulted in a complete circular *C. annuum* var. *glabriusculum* cp genome sequence (Figure 1). The genome sequence was deposited into GenBank under the accession number KR078311.

### 2.2. Features of the C. annuum var. glabriusculum Chloroplast Genome

The *C. annuum* var. *glabriusculum* cp genome is 156,817 bp in length. The GC content of the cp genome was 37.7%. The inverted repeats (IRs) had higher GC contents (43.05%) than those of the large single copy (LSC) (35.74%) or small single copy (SSC) (32.01%) regions due to the presence of GC-rich rRNA genes. The *C. annuum* var. *glabriusculum* cp genome is circular with quadripartite organization (Figure 1). The quadripartite structure includes two single copy DNA fragments, a LSC of 87,380 bp and a SSC of 17,853 bp, separated by a pair of IRs of 25,792 bp on a single circular molecule. The cp genome contains a total of 132 predicted genes (Table 1), including 87 protein-coding genes, 8 ribosomal RNA (rRNA) genes and 37 transfer RNA (tRNA) genes. Seven of these genes are duplicated in the IR regions, nine genes (Rps16, atpF, rpoC1, petB, petD, rpl16, rpl2 (IR), ndhB (IR), ndhA) and six tRNA genes contain one intron, and two genes (clpP, rps12) and one ycf (ycf3) contain two introns.

**Table 1:** General features of the *C. annuum* var. *glabriusculum* chloroplast genome.

Features	Chloroplast
Genome size (bp)	156,817
GC content (%)	37.7
Total number of genes	132
Protein coding genes	87
No. of rRNA genes	8
No. of tRNA genes	37
No. of gene duplicated in IR regions	7
Total introns	12
Single intron (gene)	9
Double introns (gene)	3
Single intron (tRNA)	6

### 2.3. Discovery of SSRs and SNPs

A total of 125 potential SSRs motifs were identified which are located mostly in the non-coding regions (Table S1), and the majority belonged to tetra-nucleotide (50%) and tri-nucleotide (26%) repeats. All other types of SSRs such as di and penta-nucleotide motifs were relatively low (25%), and the majority of tetra-nucleotide SSRs had the AAAT/AATA/ATAA motif, followed by those with the ATAA/TAAA/AAAT motif, and the remaining those with the TTTG/TTGT/TGTT, TCTT/CTTT/TTTC, and AATT/ATTA/TTAA motifs were found with similar proportion (7.2%). Two different repeats those with the TTTTA/TTTAT/TTATT, and TTATT/TATTT/ATTTT motifs were identified among penta-nucleotide SSRs. The TTC/TCT/CTT and TTA/TAT/ATT motifs were identified among the tri-nucleotide SSRs. Only, the TA/AT motif was identified as the dinucleotide SSRs (Table S1). Comparison of *C. annuum* var. *glabriusculum* cp genome sequence with the reference cp sequence of *C. annuum* revealed a total of 48 mutations (15 SNPs and 33 InDels) and 32 of these variants involving more than one nucleotide (Tables S2 and S3). Amongst the detected variants, 5 SNPs and 3 InDels were observed in the coding region of the cp genome. Amongst these SNPs and InDels, there were 43 and 5 mutations located in LSC and SSC region, respectively.

### 2.4. Discussion

Here we report the re-sequencing and assembly of a cp genome using the Illumina sequencing platform in which we recovered four contigs comprising 156,817 bp covering the entire *C. annuum* var. *glabriusculum* cp genome. Reported *Capsicum* cp genomes range in size from 156,612 to 156,781 bp, and the size of the *C. annuum* var. *glabriusculum* cp genome identified here is consistent with those reported previously in plants of the same species [18,20]. The entire cp genome of *C. annuum* var. *glabriusculum* was 36 bp longer than the reported *C. annuum* L. cp genome (GenBank accession NC_018552) and 205 bp longer than another *C. annuum* var. *glabriusculum* cp genome (GenBank accession KJ619462). Also, the SSC and IR regions of *C. annuum* var. *glabriusculum* were 3 and 9 bp longer, respectively, and the LSC region was 14 bp shorter and 167 bp longer, respectively, than those of the previously reported cp genomes. The average GC content in the *C. annuum* cp genome is 37.7%, similar to other *Capsicum* species. The data generated using the Illumina platform covered a greater depth (188×) of the cp genome whereas, in the previous studies cp genome sequence coverage was not reported and were able to resolve the ambiguities present in the GS-FLX pyrosequencing. Thus, the data from the cp assembly reported here supports previous findings that Illumina can produce high quality sequence assemblies covering a greater genome depth [21].

The organization and gene order of the *Capsicum* cp genome exhibited the general cp genome structure of angiosperms [22]. The *Capsicum* cp genome contained 132 genes (Table 2), of which there were 8 rRNA genes, 37 tRNA genes, 21 ribosomal subunit genes (12 small subunit and 9 large subunit) and 4 DNA-directed RNA polymerase genes. Forty-six genes were involved in photosynthesis, of which 11 encoded subunits of the NADH-oxidoreductase, 7 for photosystem I, 15 for photosystem II, 6 for the cytochrome b6/f complex, 6 for different subunits of ATP synthase and 1 for the large chain of ribulose bisphosphate carboxylase. Five genes were involved in different functions, and three genes were of unknown function. As shown in Figure 1 and Table 2, genome organization appeared to be more conserved with unique gene sequences, as discovered previously in *Capsicum* species [18,19,20]. However, in this newly determined cp genome, we found 132 predicted genes and size variations were observed in the IR and LSC regions. A total of 125 cpSSRs markers were identified in 156.8 kb sequence of the *Capsicum* chloroplast genome. The observed frequency of SSRs was approximately 1/1.25 kb of chloroplast genome. More interestingly, the cpSSRs were only observed in the non-coding region of the cp genome. Similarly, most of the SNPs and InDels in the cp genome present in intergenic region, and only 8 variants were located in genic region (Tables S2 and S3).

**Table 2:** Genes present in the *C. annuum* var. *glabriusculum* chloroplast genome.

Gene Products of *Capsicum annuum* var. *glabriusculum*	
Photosystem I	psaA, B, C, I, J, ycf3 ^2^, ycf4
Photosystem II	psbA, B, C, D, E, F, H, I, J, K, L, M, N, T, Z
Cytochrome b6/f	petA, B ^1^, D ^1^, G, L, N
ATP synthase	atpA, B, E, F ^1^, H, I
Rubisco	rbcL
NADH oxidoreductase	ndhA ^1^, B ^1,3^, C, D, E, F, G, H, I, J, K
Large subunit ribosomal proteins	rpl2 ^1,3^, 14, 16 ^1^, 20, 22, 23 ^3^, 32, 33, 36
Small subunit ribosomal proteins	rps2, 3, 4, 7 ^3^, 8, 11, 12 ^2,3,4^, 14, 15, 16 ^1^, 18, 19
RNA polymerase	rpoA, B, C1 ^1^, C2
Unknown function protein coding gene	ycf1 ^3^, 2 ^3^, 15 ^3^
Other genes	accD, ccsA, cemA, clpP ^2^, matK
rRNAs	rrn16 ^3^, 23 ^3^, 4.5 ^3^, 5 ^3^
tRNAs	trnA-UGC ^1,3^, trnC-GCA, trnD-GUC, trnE-UUC, trnF-GAA, trnG-UCC ^1^, trnG-GCC, trnH-GUG, trnI-CAU ^3^, trnI-GAU ^1,3^, trnK-UUU ^1^, trnL-UAA ^1^, trnL-UAG, trnL-CAA ^3^, trnfM-CAU, trnM-CAU, trnN-GUU ^3^, trnP-UGG, trnQ-UUG, trnR-ACG ^3^, trnR-UCU, trnS-GCU, trnS-GGA, trnS-UGA, trnT-GGU, trnT-UGU, trnV-UAC ^1^, trnV-GAC ^3^, trnW-CCA, trnY-GUA

^1^ Genes containing a single intron; ^2^ Genes containing two introns; ^3^ Two gene copies in IR; ^4^ Trans-splicing gene.

## 3. Experimental Section

### 3.1. Sampling and DNA Extraction

Sample (accession No. IT158289) was obtained from the National Agrobiodiversity Center, Rural Development Administration, Korea. Fresh leaves were collected from 40-day-old seedlings, and DNA was extracted to construct cp DNA libraries.

### 3.2. Library Preparation and Sequencing

An Illumina paired-end cp DNA library (average insert size of 500 bp) was constructed using the Illumina TruSeq library preparation kit following the manufacturer’s instructions. The libraries were sequenced with 2 × 300 bp on the MiSeq instrument at LabGenomics (http://www.labgenomics.co.kr/).

### 3.3. Chloroplast Genome Assembly

Prior to cp *de novo* assembly, low quality sequences (quality score < 20; Q20) were filtered out, and the remaining high quality reads were assembled using the CLC Genome Assembler (version beta 4.6, CLC Inc. Aarhus, Denmark) with a 200-600-bp overlap size. Cp contigs were selected from the initial assembly by performing a BLAST search against known cp sequences (GenBank accession NC_018552). The selected contigs were oriented to construct the complete cp genome structure. Ambiguous nucleotides or gaps were corrected manually to build the complete cp genome.

### 3.4. Gene Annotation

The web-based program Dual OrganellarGenoMe Annotator (DOGMA, http://dogma.ccbb.utexas.edu/) was used to annotate the assembled genome using default parameters to predict protein coding, tRNA and rRNA genes. Subsequently, BLASTN was used to further identify intron-containing gene positions by searching a published cp genome database. A cp gene map was constructed using the OrganellarGenomeDRAW software (OGDRAW, http://ogdraw.mpimp-golm.mpg.de).

### 3.5. Discovery of SNPs and SSRs

Sputnik (http://espressosoftware.com/pages/sputnik.jsp) software was used to find the SSR markers present in the cp genome of *C. annuum* var. *glabriusculum*. It uses a recursive algorithm to search for repeats with length between 2 and 5, and finds perfect, compound and imperfect repeats. Sputnik has been applied for SSR identification in many species including Arabidopsis and barley [23]. To identify SNP and INDEL variants in *C. annuum* var. *glabriusculum* cp genome, we used BWA [24] and Samtools [25] software. More detailed method and algorithm are descripted in Li (2012) [26].

## 4. Conclusions

The cp genome sequences of *Capsicum* species, such as *C. annuum* var. *glabriusculum,* *C. annuum* var. *annuum* and *C. annuum* L., have been reported previously; however, information on cp gene content is limited. The complete cp genome sequence of *Capsicum* pepper (*C. annuum* var. *glabriusculum*) reported here enhances the genomic information for *C. annuum* and contributes to the study of germplasm diversity. These data represent a valuable source of markers for future studies on *Capsicum* populations. Moreover, the complete cp genome sequence also provides data on functional protein variability in the cp.

## Supplementary Materials

Supplementary materials can be accessed at: http://www.mdpi.com/1420-3049/20/07/13080/s1.

## References

[1] Rodriguez-Ezpeleta N., Brinkmann H., Burey S.C., Roure B., Burger G., Loffelhardt W., Bohnert H.J., Philippe H., Lang B.F. (2005). Monophyly of primary photosynthetic eukaryotes: Green plants, red algae, and glaucophytes. Curr. Biol..

[2] Tetlow I.J., Rawsthorne S., Raines C., Emes M.J., Moller S.G. (2009). Plastid metabolic pathways. Annual Plant Reviews, Plastids.

[3] Ohyama K., Fukuzawa H., Kohchi T., Shirai H., Sano T., Sano S., Umesono K., Shiki Y., Takeuchi M., Chang Z. (1986). Chloroplast gene organization deduced from complete sequence of liverwort marchantia polymorpha chloroplast DNA. Nature.

[4] Shinozaki K., Ohme M., Tanaka M., Wakasugi T., Hayashida N., Matsubayashi T., Zaita N., Chunwongse J., Obokata J., Yamaguchi-Shinozaki K. (1986). The complete nucleotide sequence of the tobacco chloroplast genome: Its gene organization and expression. EMBO J..

[5] http://chloroplast.ocean.washington.edu/cpbase.

[6] Sugiura M., Hirose T., Sugita M. (1998). Evolution and mechanism of translation in chloroplasts. Ann. Rev. Genet..

[7] Daniell H., Lee S.B., Grevich J., Saski C., Quesada-Vargas T., Guda C., Tomkins J., Jansen R.K. (2006). Complete chloroplast genome sequences of solanum bulbocastanum, solanum lycopersicum and comparative analyses with other solanaceae genomes. Theor. Appl. Genet..

[8] Leseberg C.H., Duvall M.R. (2009). The complete chloroplast genome of coix lacryma-jobi and a comparative molecular evolutionary analysis of plastomes in cereals. J. Mol. Evol..

[9] Moscone E.A., Scaldaferro M.A., Grabiele M., Cecchini N.M., Sanchez Garcia Y., Jarret R., Davina J.R., Ducasse D.A., Barboza G.E., Ehrendorfer F. (2007). The evolution of chili peppers (*Capsicum*-Solanaceae): A cytogenetic perspective. Acta Hortic..

[10] Eshbaugh W.H., Janick J., Simon J.E. (1993). History and exploitation of a serendipitous new crop discovery. New Crops.

[11] Pozzobon M.T., Schifino-Wittmann M.T., Bianchetti L.D.B. (2005). Chromosome numbers in wild and semidomesticated Brazilian *Capsicum* L. (Solanaceae) species: Do x = 12 and x = 13 represent two evolutionary lines?. Bot. J. Linn. Soc..

[12] Kumar S., Kumar R., Singh J., Peter K.V. (2006). Cayenne/American pepper (*Capsicum* species). Handbook of Herbs and Spices.

[13] Dong W., Liu J., Yu J., Wang L., Zhou S. (2012). Highly variable chloroplast markers for evaluating plant phylogeny at low taxonomic levels and for DNA barcoding. PLoS ONE.

[14] Hollingsworth P.M., Graham S.W., Little D.P. (2011). Choosing and using a plant DNA barcode. PLoS ONE.

[15] Group C.P.W., Hollingsworth P.M., Forrest L.L., Spouge J.L., Hajibabaei M., Ratnasingham S., van der Bank M., Chase M.W., Cowan R.S., Erickson D.L. (2009). A DNA barcode for land plants. Pro. Natl. Acad. Sci. USA.

[16] Selvaraj D., Sarma R., Shanmughanandhan D., Srinivasan R., Ramalingam S. (2015). Evaluation of DNA barcode candidates for the discrimination of the large plant family Apocynaceae. Plant Syst. Evol..

[17] Spouge J.L., Mariño-Ramírez L. (2012). The practical evaluation of DNA barcode efficacy. Methods Mol. Biol..

[18] Zeng F.C., Gao C.W., Gao L.Z. (2014). The complete chloroplast genome sequence of American bird pepper (*Capsicum annuum* var. *glabriusculum*). Mitochondrial DNA.

[19] Sebastin R., Young-Ah J., Jung-Ro L., Gi-An L., Kyung Jun L., Gyu-Taek C., Kyung-Ho M., Sok-Young L., Jong-Wook C. (2015). The complete chloroplast genome sequence of Korean landrace “subicho” pepper (*Capsicum annuum* var. *annuum*). Plant Breed. Biotechnol..

[20] Jo Y.D., Park J., Kim J., Song W., Hur C.G., Lee Y.H., Kang B.C. (2011). Complete sequencing and comparative analyses of the pepper (*Capsicum*
*annuum* L.) plastome revealed high frequency of tandem repeats and large insertion/deletions on pepper plastome. Plant Cell Rep..

[21] Wu Z.H., Gui S.T., Quan Z.W., Pan L., Wang S.Z., Ke W.D., Liang D.Q., Ding Y. (2014). A precise chloroplast genome of *Nelumbo nucifera* (Nelumbonaceae) evaluated with Sanger, Illumina Miseq, and Pacbio RS II sequencing platforms: Insight into the plastid evolution of basal eudicots. BMC Plant Biol..

[22] Sugiura M. (1992). The chloroplast genome. Plant Mol. Biol..

[23] Cardle L., Ramsay L., Milbourne D., Macaulay M., Marshall D., Waugh R. (2000). Computational and experimental characterization of physically clustered simple sequence repeats in plants. Genetics.

[24] Li H., Durbin R. (2009). Fast and accurate short read alignment with burrows-wheeler transform. Bioinformatics.

[25] Li H., Handsaker B., Wysoker A., Fennell T., Ruan J., Homer N., Marth G., Abecasis G., Durbin R., Proc G.P.D. (2009). The sequence alignment/map format and samtools. Bioinformatics.

[26] Li H. (2012). Exploring single-sample SNP and INDEL calling with whole-genome *de novo* assembly. Bioinformatics.

