# Supplementary material for: The Complete Chloroplast Genome of Capsicum annuum var. glabriusculum Using Illumina Sequencing
Source: Molecules. 2015 Jul 20;20(7):13080–8. doi: 10.3390/molecules200713080 (PMC6332240; doi:10.3390/molecules200713080)
Supplement: Supplementary file 1 [file molecules-20-13080-s001.pdf]

Supplementary Information

Table S1. SSR candidates of *Capsicum annuum* var. *glabriusculum* chloroplast genome.

Type of SSR	SSR Abundances	Percentage Abundance (%)
Dinucleotide		
TA/AT	9	7.2
Trinucleotide		
TTC/TCT/CTT	9	7.2
TTA/TAT/ATT	23	18.4
Tetranucleotide		
TTTG/TTGT/TGTT	9	7.2
TCTT/CTTT/TTTC	9	7.2
ATAA/TAAA/AAAT	16	12.8
AATT/ATTA/TTAA	9	7.2
AAAT/AATA/ATAA	19	15.2
Pentanucleotide		
TTTTA/TTTAT/TTATT	11	8.8
TTATT/TATTT/ATTTT	11	8.8
Total	125	100

Table S2. SNP markers of *Capsicum annuum* var. *glabriusculum* chloroplast genome.

No.	REF (*C. annuum*)	ALT (*C. annuum* var. *glabriusculum*)	Coding Region	QUAL	Region
1	T	C	non-coding region	222	LSC
2	A	T	non-coding region	222	LSC
3	G	T	non-coding region	222	LSC
4	G	A	non-coding region	222	LSC
5	C	A	non-coding region	222	LSC
6	T	A	non-coding region	222	LSC
7	A	G	non-coding region	222	LSC
8	G	C	Gene (petA)	222	LSC
9	A	G	Gene (petA)	222	LSC
10	C	A	Gene (petA)	222	LSC
11	C	A	Gene (petA)	222	LSC
12	A	C	Gene(psbF)	15.1	LSC
13	A	T	non-coding region	222	LSC
14	A	T	non-coding region	222	SSC
15	T	G	non-coding region	222	SSC

Table S3. INDEL markers of *Capsicum annuum* var. *glabriusculum* chloroplast genome.

No.	REF (*C. annuum*)	ALT (*C. annuum* var. *glabriusculum*)	Coding Region	QUAL	Region
1	ATTTTTTTTT	ATTTTTTTTTT	non-coding region	61.5	LSC
2	CTTTTTTTT	CTTTTTTTTT,CTTTTTTTTTT	non-coding region	91.5	LSC
3	TAAAAAAAA	TAAAAAAAAA,TAAAAAAAAAA	non-coding region	84.5	LSC
4	CTTTTT	CTTTTTT	non-coding region	207	LSC
5	TAAAAAA	TAAAAAAA,TAAAAAAAAAA	non-coding region	158	LSC
6	GAAAAAAAAAAAAAAAA	GAAAAAAAAAAAAAAAAA,GAAAAAAAAAAAAAAAAAA,GAAAAAAAAA	non-coding region	3.24	LSC
7	CTTTTTT	CTTTTTTT,CTTTTTTTT	non-coding region	134	LSC
8	CTTTTTTTTTTTT	CTTTTTTTTTTTTT,CTTTTTTTTTTTTTT	non-coding region	11.8	LSC
9	TAAAAAAA	TAAAAAAAA,TAAAAAAAAA	non-coding region	121	LSC
10	GTTTTTTT	GTTTTTT	non-coding region	129	LSC
11	GTTTTTTTTT	GTTTTTTTTTT,GTTTTTTTTTTT,GTTTTTTTTTTTT	non-coding region	74.5	LSC
12	TCAACTCATTTTA	T	non-coding region	214	LSC
13	ATTTTT	ATTTTTT	non-coding region	195	LSC
14	CAAAAAAAAAA	CAAAAAAAAAAA,CAAAAAAAAAAAA,CAAAAAAAA	non-coding region	56.5	LSC
15	ATTTTTTTTT	ATTTTTTTTTT,ATTTTTTTTTTT	non-coding region	68.5	LSC
16	TAAAAAAAAAA	TAAAAAAAAAAA,TAAAAAAAAAAAA	non-coding region	49.5	LSC
17	GAAAAAA	GAAAAAAA	non-coding region	148	LSC
18	ATTTTTTTTT	ATTTTTTTT	non-coding region	96.5	LSC
19	CAAAAAAAAAA	CAAAAAAAAAAA,CAAAAAAAAAAAA	non-coding region	48.5	LSC
20	AATCAAAGTAAATAAGAT	AAT	non-coding region	217	LSC
21	TAAAAAAAA	TAAAAA	non-coding region	214	LSC
22	TT	TTCTTTTATAT	non-coding region	214	LSC
23	ATTTTTTTTT	ATTTTTTTTTT,ATTTTTTTTTTT	non-coding region	82.5	LSC
24	AGACTCCGGTAA	AGACTCCGGTAACGACTCCGGTAA	Gene (rpl20)	218	LSC
25	GTTTTTTTT	GTTTTTTTTT,GTTTTTTTTTT	non-coding region	94.5	LSC
26	GAAAAAAA	GAAAAAA	non-coding region	70.5	LSC
27	GAAAAAAAA	GAAAAAAAAA,GAAAAAAAAAA,GAAAAAAAAAAA	non-coding region	90.5	LSC
28	CTTTTTTTTTTT	CTTTTTTTTTTTT,CTTTTTTTTTTTTT	non-coding region	39.5	LSC
29	CTTTT	CTTTTT	non-coding region	214	LSC
30	ATTCTTATTTTTT	ATTATTTTTT	Gene (rps19)	214	LSC
31	GTTTTTTTTT	GTTTTTTTTTTT	Gene (rpl32)	29.5	SSC
32	TAAAAAAAAA	TAAAAAAAAAA,TAAAAAAAAAAA	non-coding region	75.5	SSC
33	TCCCCC	TCCCCCC	non-coding region	187	SSC
